# Matriptase and prostasin are expressed in human skin in an inverse trend over the course of differentiation and are targeted to different regions of the plasma membrane

**DOI:** 10.1242/bio.019745

**Published:** 2016-08-19

**Authors:** Chih-Hsin Lai, Shun-Cheng Chang, Yen-Ju Chen, Yi-Jie J. Wang, Ying-Jun J. Lai, Hsiang-Hua D. Chang, Eric B. Berens, Michael D. Johnson, Jehng-Kang Wang, Chen-Yong Lin

**Affiliations:** 1 Department of Dentistry Renai Branch, Taipei City Hospital, Taipei 114, Taiwan; 2 Graduate Institute of Medical Sciences, National Defense Medical Center, Taipei 114, Taiwan; 3 Division of Plastic Surgery, Department of Surgery, Shuang-Ho Hospital. School of Medicine, College of Medicine, Taipei Medical University, Taipei 110, Taiwan; 4 Department of Biochemistry, National Defense Medical Center, Taipei 114, Taiwan; 5 School of Medicine, National Defense Medical Center, Taipei 114, Taiwan; 6 Lombardi Comprehensive Cancer Center, Department of Oncology Georgetown University Washington DC 20057, USA

**Keywords:** Matriptase, Prostasin, Skin

## Abstract

Matriptase and prostasin, acting as a tightly coupled proteolytic cascade, were reported to be required for epidermal barrier formation in mouse skin. Here we show that, in human skin, matriptase and prostasin are expressed with an inverse pattern over the course of differentiation. Matriptase was detected primarily in epidermal basal keratinocytes and the basaloid cells in the outer root sheath of hair follicles and the sebaceous gland, where prostasin was not detected. In contrast, prostasin was detected primarily in differentiated cells in the epidermal granular layer, the inner root sheath of hair follicles, and the sebaceous gland, where matriptase expression is negligible. While co-expressed in the middle stage of differentiation, prostasin was detected as polarized patches, and matriptase at intercellular junctions. Targeting to different subcellular localizations is also observed in HaCaT human keratinocytes, in which matriptase was detected primarily at intercellular junctions, and prostasin primarily on membrane protrusion. Furthermore, upon induction of zymogen activation, free active prostasin remains cell-associated and free active matriptase is rapidly shed into the extracellular milieu. Our data suggest that matriptase and prostasin likely function as independent entities in human skin rather than as a tightly coupled proteolytic cascade as observed in mouse skin.

## Abstract (precis)

**Summary:** The inverse expression pattern between matriptase and prostasin during differentiation in human skin demonstrated here provides a stark contrast to their close functional link suggested from mouse models.

## INTRODUCTION

The tightly coupled proteolytic cascade, composed of the type 2 transmembrane serine protease matriptase and the glycosylphosphatidylinositol (GPI)-anchored serine protease prostasin, plays pivotal roles in the formation of the epidermal barrier in mouse skin (Leyvraz et al., 2005; List et al., 2002). Genetic ablation of matriptase or prostasin causes severe impairment of epidermal barrier function leading to neonatal death for matriptase and prostasin knockout mice. Both serine proteases may directly participate in the programs involved in epidermal barrier formation in terminally differentiated mouse keratinocytes, as both proteases are expressed predominantly in the outermost layer of viable epidermal keratinocytes in the mouse (Netzel-Arnett et al., 2006). Matriptase appears to function as an upstream activator for prostasin, converting the zymogen form into the active protease, since it has been shown that active prostasin is not detectable in the skin of matriptase knockout mice. Prostasin may be the only downstream protease responsible for mediating matriptase function in the formation of mouse epidermal barrier, given the almost identical epidermal defects observed in the skin of matriptase and prostasin knockout mice.

The functional partnership between matriptase as an upstream activator and prostasin as a downstream substrate in mouse keratinocytes has been also observed in HaCaT immortalized human keratinocytes, in which induction of matriptase zymogen activation also rapidly leads to prostasin zymogen activation, and matriptase is required for acid-induced activation of prostasin zymogen (Chen et al., 2010b). Studies using HaCaT cells further reveal that the epidermal matriptase-prostasin proteolytic cascade is under tight control by hepatocyte growth factor activator inhibitor (HAI)-1, a Kunitz-type serine protease inhibitor, which rapidly inhibits not only matriptase but also prostasin, following the induction of zymogen activation of both serine proteases. The evidence for a physiologically relevant *in vivo* partnership between these proteases and HAI-1 has been bolstered by the purification and identification of matriptase-HAI-1 and prostasin-HAI-1 complexes from human milk and other body fluids (Lai et al., 2016; Lin et al., 1999; Wang et al., 2009).

In spite of the above-mentioned functional relationship, there seems to be some evolutionary divergence between human and mouse with regard to matriptase, which is expressed primarily in the basal and spinous layers of the human epidermis and participates in the control of epidermal proliferation and early differentiation (Chen et al., 2013a). The pattern of human matriptase expression *in vivo* with differentiation-associated down-regulation has been also observed in human hair follicles, sebaceous glands (Wu et al., 2013), and replicated in the organotypic skin raft model (Chen et al., 2013a). This is in stark contrast to the differentiation-associated up-regulation of matriptase observed in mouse skin. Similar differentiation-associated up-regulation of matriptase expression has also been observed in mouse hair follicles, with the exception that matriptase expression is present in the proliferative matrix cells of mouse hair follicles (List et al., 2007). This evolutional divergence in the pattern and potentially function of matriptase in human versus mouse physiology may in part explain the much reduced severity of the epidermal defects observed in patients with matriptase mutations, including the ones resulting in loss of matriptase expression (Alef et al., 2009). Given the different role apparently played by matriptase in human versus mouse skin biology it seems reasonable that the role of prostasin, and the functional partnership between matriptase and prostasin observed in mouse skin, may not be the same in human skin. In the current study, we investigate the functional relationship between human matriptase and prostasin by focusing on their tissue distribution profile *in vivo* in the human epidermis, hair follicles, and sebaceous gland, their subcellular localization and the fates of the active enzymes *in vitro* in HaCaT human keratinocytes. Our study reveals that in spite of the close functional link established with mouse models, human matriptase and human prostasin may function as independent entities and participate in different stages of differentiation in the three compartments of human skin.

## RESULTS

### Differential distribution of matriptase versus prostasin in human epidermis, hair follicles and sebaceous gland

The epidermis, hair follicle and sebaceous gland share a similar differentiation scheme with layers of cells at different stages of differentiation. The distribution profiles of matriptase, prostasin and their cognate inhibitor HAI-1 were determined by immunohistochemistry (IHC) and compared in the three tissues. The specimens examined in the current study were obtained from more than 100 patients with a variety of skin diseases. Our conclusions are based on the reproducible IHC staining patterns in the skin specimens from more than 20 patients with those skin diseases, which do not affect the histological morphology of the epidermis; these skin diseases including melanocytic nevus, skin tag, unruptured epidermal cyst, etc. The specimens examined were taken from the periphery of these skin lesions. The same IHC staining patterns were also observed in a normal skin specimen from a healthy donor.

**Fig. 1.**
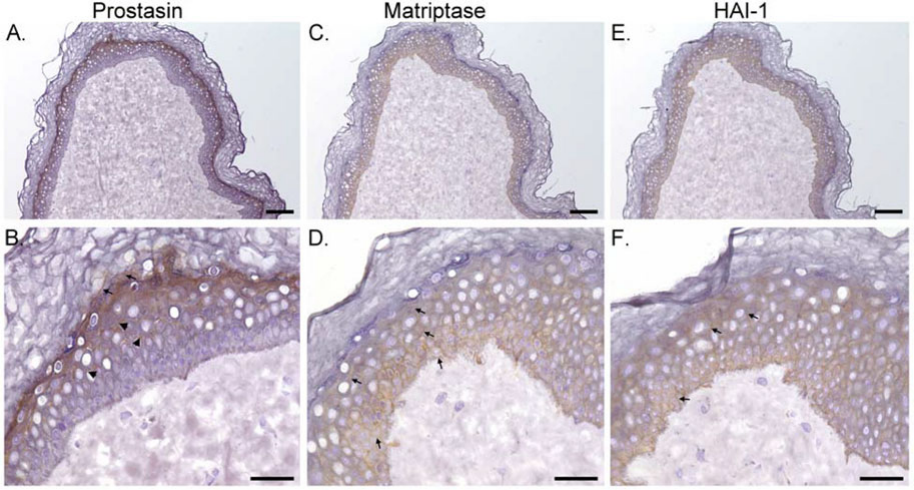
**Inverse pattern of prostasin versus matriptase expression during the course of epidermal differentiation.** Human skin tissue sections were immunostained for prostasin (A,B), matriptase (C,D), and HAI-1 (E,F). Other sections were also stained with a non-specific mouse IgG antibody as a negative control (data not shown). Representative examples of the staining observed are presented. Cell nuclei were lightly counterstained with hematoxylin. Scale bars: 100 µm in A, C, and E; 40 µm in B, D, and F.

**Fig. 2.**
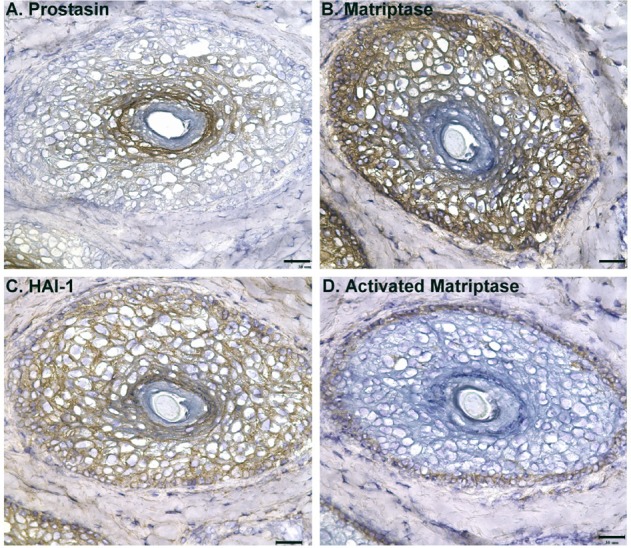
**Expression of prostasin, matriptase, activated matriptase, and HAI-1 in human hair follicles.** Cross sections of human hair follicles were immunostained with the prostasin-specific mAb YL11 (A), matriptase-specific mAb M24 (B), the HAI-1-specific mAb M19 (C) or the activated matriptase-specific mAb M69 (D) and counterstained with hematoxylin. Sections were also stained with a non-specific mouse IgG antibody as a negative control (data not shown). Representative examples of the staining observed are presented. Scale bars: 30 µm.

Human epidermis is composed of distinct layers, including the basal, spinous, and granular layers, which represent different stages of keratinocyte differentiation, from proliferation, through early differentiation, to terminal differentiation. As shown in Fig. 1A, prostasin was clearly detected as a thin brown thread along the transition border between the layers with viable cells and the layers with dead cells. At higher magnification (Fig. 1B), the staining indicates the presence of prostasin at high levels on the granular layer keratinocytes. While diffuse staining of prostasin was observed in this epidermal layer, some prostasin is likely localized at the intercellular junction (Fig. 1B, arrow). In the upper and middle spinous layers, which are adjacent to the granular layers, prostasin was clearly detected beneath the characteristic intercellular bridges and always in a polarized manner (Fig. 2B, arrow head). The cells of the basal layer and the adjacent lower spinous layers appear to be devoid of prostasin staining.

The matriptase distribution profile in the epidermis is in marked contrast to that for prostasin. Matriptase appears to be expressed by all cells in the viable epidermal layers except those in the granular layer, which is seen as a thin blue thread between the viable cells and dead cells (Fig. 1C). At higher magnification, matriptase staining can be seen at the intercellular junctions in the basal and spinous layers but not in the granular layer (Fig. 1D, arrows). HAI-1 was detected in all three of the epidermal layers, again, primarily at the cell-cell junctions (Fig. 1E,F, arrows). The differential distributions of the three proteins and the lack of staining with the mouse IgG negative control (data not shown) demonstrate the specificity of the IHC. The distribution profile observed implies that so far as matriptase contributes to epidermal biology, it is most likely via regulation of keratinocyte proliferation and early differentiation, with its function becoming less important during the later stages of epidermal differentiation. Conversely, the prostasin distribution pattern is more consistent with a role in the later stages of differentiation, whereas the GPI-anchored protease might participate in the differentiation of spinous keratinocytes prior to their conversion into granular cells. The lack of prostasin in the basal keratinocytes suggests that matriptase contributes to the function of basal keratinocytes through a mechanism that does not depend on its activation of prostasin as the downstream substrate. Furthermore, the absence of matriptase in the granular layer keratinocytes suggests that prostasin participates in late-stage differentiation in the absence of any control by matriptase as an upstream activator. Thus, any functional linkage between matriptase and prostasin that may exist would seem to be limited to some spinous-layer keratinocytes, in which both membrane-associated proteases are co-expressed. In contrast to the stage-dependent expression profiles for matriptase and prostasin, HAI-1 is expressed in all of the viable epidermal layers. This suggests that this Kunitz-type inhibitor can participate in distinct processes of epidermal differentiation through the regulation of different target proteases in a stage-dependent manner in human epidermis.

**Fig. 3.**
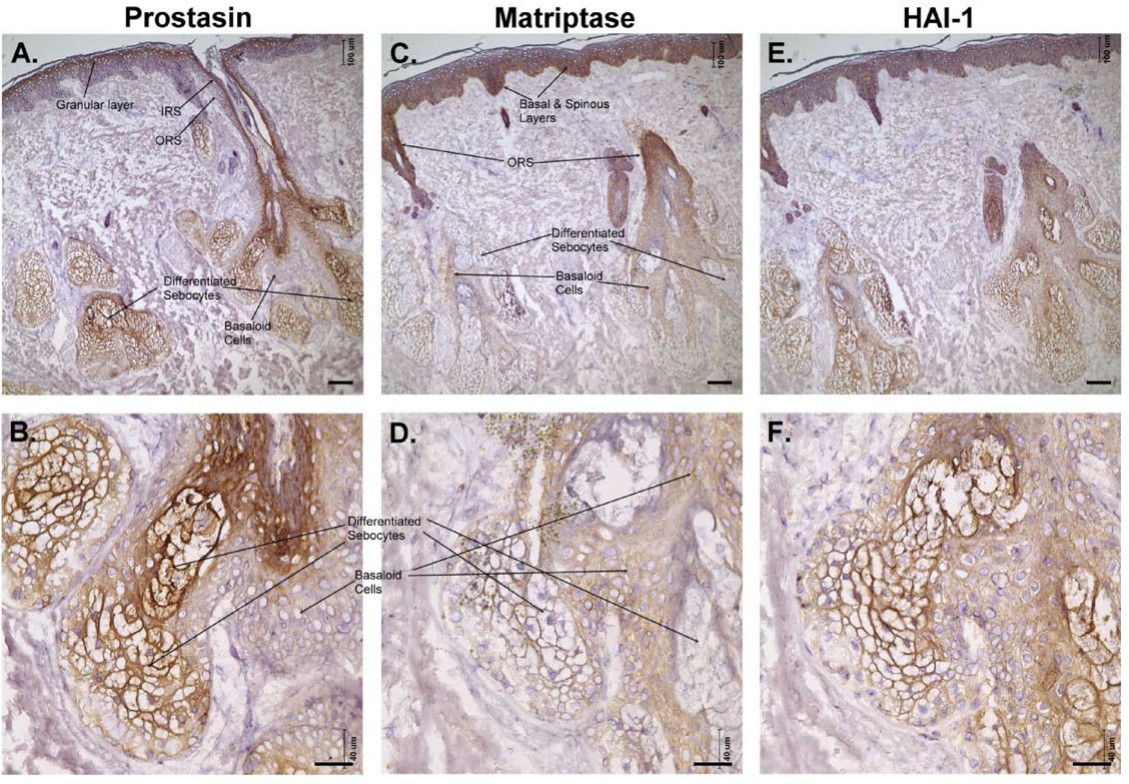
**Expression of prostasin, matriptase and HAI-1 in human skin.** Human skin tissue sections containing the epidermis, hair follicles, and sebaceous glands were immunostained with the prostasin mAb YL11 (prostasin; A,B), matriptase-specific mAb M24 (matriptase; C,D), or the HAI-1-specific mAb M19 (HAI-1; E,F), and counterstained with hematoxylin. Other sections were also stained with a non-specific mouse IgG antibody as a negative control (data not shown). Representative examples of the staining observed are presented. The different regions of the skin section are as indicated, with the sebaceous glands shown at higher magnification in the lower panels. Scale bars: 100 µm in A, C, and E; 40 µm in B, D, and F.

In addition to the epidermis, the skin contains hair follicles and sebaceous glands. The pilosebaceous unit is histologically connected with and resembles the epidermis in that it is composed of proliferating, differentiating, and differentiated cells. The inverse distribution profiles for matriptase and prostasin during the course of differentiation, and the broad distribution of HAI-1 expression observed in the epidermis were also observed in hair follicles (Figs 2 and 3) and sebaceous glands (Fig. 3). In a cross-section of hair follicles (Fig. 2A) prostasin staining was present at high levels on the inner root sheet (IRS), which is composed of differentiated cells, with the intensity of staining gradually decreasing and eventually becoming negative in the outer root sheet (ORS), which contains active cells, providing a protective encasement for the growing hair shaft. In the reverse trend, matriptase staining was at high levels on the outer layers of the ORS, with the intensity of staining decreasing through the inner layers of the ORS and eventually becoming negative in the IRS (Fig. 2B). The broad distribution of HAI-1 in both active and differentiated cells is again seen in hair follicles with positive staining of HAI-1 observed both in the ORS and the IRS (Fig. 2C).

Matriptase is synthesized as a zymogen with only very limited intrinsic activity and only acquires full enzymatic activity upon cleavage at the canonical activation motif, a process which causes localized conformational changes in its serine protease domain (Benaud et al., 2001). We have generated a group of matriptase mAbs, including the mAb M69, which are believed to recognize these localized conformational changes and can distinguish between activated matriptase (to which they bind) and the zymogen form of the enzyme (to which they do not bind). The mAb M69 can be used in IHC to assess the status of matriptase zymogen activation (Chen et al., 2011; Wang et al., 2009). Among the matriptase-expressing cells in the ORS, the activated form of matriptase was detected only in the cells in the uttermost layer (Fig. 2D), which are physically distant from the prostasin-expressing cells in the IRS.

This consistent pattern with the inverse distribution of prostasin versus matriptase both in the epidermis and the hair follicles was particularly clear in skin areas where the upper part of the hair follicle blends with the surface epidermis (Fig. 3). In Fig. 3 the positive staining for prostasin in the granular layer of epidermis expanded into the IRS of hair follicles (Fig. 3A), and the positive staining of matriptase in the basal layer and the lower spinous layer in the epidermis expanded into the ORS of hair follicles (Fig. 3C).

The sebaceous gland arises as an outgrowth of the ORS of the hair follicle and is composed of undifferentiated basaloid cells at the periphery, and partially and fully differentiated sebocytes in the interior. Prostasin was detected at high levels at the differentiated sebocytes (Fig. 3A,B), in which matriptase staining was negative (Fig. 3C,D). In contrast, the peripheral basaloid cells show very weak prostasin staining (Fig. 3A,B) and are positive for matriptase (Fig. 3C,D). The broad distribution of HAI-1 throughout the process of differentiation is observed again in the sebaceous gland with both the basaloid and differentiated cells staining for HAI-1 (Fig. 3E,F).

### Matriptase and prostasin are generally present in different subdomains of the plasma membrane in HaCaT human keratinocytes

**Fig. 4.**
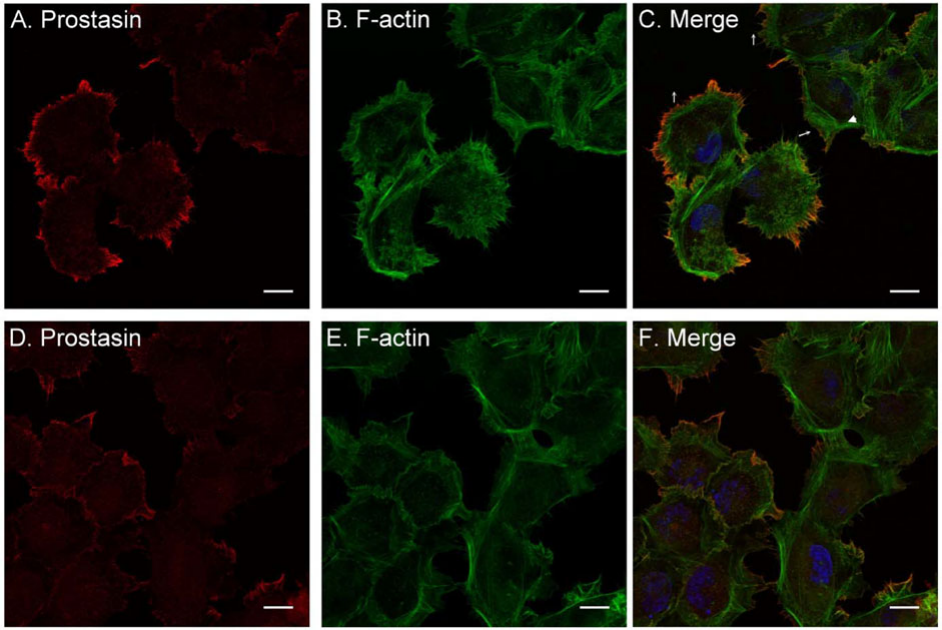
**Prostasin is localized primarily at membrane protrusions in HaCaT human keratinocytes.** The subcellular localization of prostasin (A,D; red) in HaCaT human keratinocytes were analyzed by immunofluorescent staining with the prostasin mAb YL11. The cells were also stained for F-actin using Alexa 488-labeled phalloidin (B,E; green) and nuclei using DAPI (blue), as a counterstain. The staining is also presented as merged false-color images (C,F). Scale bars: 20 µm.

**Fig. 5.**
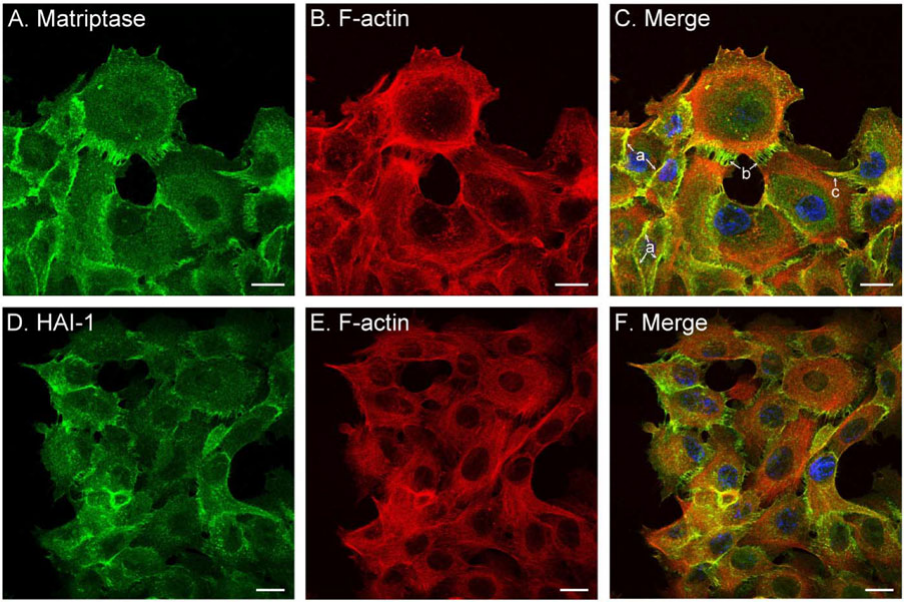
**Matriptase and HAI-1 are primarily expressed at the cell-cell junctions.** The subcellular localizations of matriptase (A; green) and HAI-1 (D; green) in HaCaT human keratinocyte cultures were analyzed by immunofluorescent staining with the matriptase-specific mAb M24 and HAI-1-specific mAb M19. The cells were also stained for F-actin using Alexa 594-labeled phalloidin (B,E; red) and nuclei using DAPI (blue), as a counterstain. The staining is also presented as merged false-color images (C,F). The staining of matriptase at different types of cell-cell contacts is as indicated. Scale bars: 20 µm.

Although matriptase and prostasin are co-expressed in the intermediate stages of differentiation, they appear to be targeted to different subdomains of the plasma membrane. This difference was initially noticed in the IHC staining, with matriptase being more or less homogenously distributed at the cell-cell junctions (Fig. 1D), whereas prostasin staining appears to be focused on one side of the cells (Fig. 1B). To pursue this observation further we next investigate the relative subcellular localization of matriptase and prostasin using HaCaT human keratinocytes, which naturally express both membrane-associated serine proteases. Consistent with their known membrane association, immunocytochemical staining revealed the presence of both prostasin (Fig. 4) and matriptase (Fig. 5) on the plasma membrane, along with the cognate inhibitor HAI-1 (Fig. 5). While both proteins are present on plasma membrane, however, they appear to be targeted to different subdomains of the plasma membrane. The most prominent staining for prostasin was observed on membrane protrusions in which prostasin is colocalized with F-actin in both filapodium and lamellipodium at the leading edges (Fig. 4A-C). Prostasin was also detected at cell-cell contacts, but at lower levels than that at the cell periphery and not in contact with neighboring cells (Fig. 4D-F). In contrast, matriptase was detected predominantly at the cell-cell contacts (Fig. 5A-C), including well-formed intercellular junctions (Fig. 5C, arrow a), areas where the assembly or disassembly of cell-cell junctions appears to be happening (Fig. 5C, arrow b), and where one cell is overlapping neighboring cells (Fig. 5C, arrow c). In addition to the cell-cell contacts, matriptase was also occasionally detected on the cell periphery in the absence of contact with neighboring cells, but at much lower levels. The distribution of HAI-1 in the HaCaT cells (Fig. 5D-F) is very similar to that of matriptase, consistent with their exceptionally tight functional partnership. The targeting of these proteins to different subdomains of plasma membrane suggests that matriptase and prostasin are under the control of different regulatory mechanisms and are involved in different cellular processes.

### Active prostasin is retained by human keratinocytes whereas active matriptase is shed into the extracellular milieu

Both matriptase and prostasin are synthesized as zymogens and acquire their full enzymatic activity only after zymogen activation, a process that can be induced by transiently exposing HaCaT cells to a mildly acidic buffer. The fate of the nascent active matriptase in HaCaT human keratinocytes has been well characterized in our previous studies with a vast majority of active matriptase being rapidly inhibited by HAI-1 and remaining cell-associated. A small proportion of nascent active matriptase survives HAI-1 inhibition by rapidly shedding to the extracellular milieu (Chen et al., 2010b; Chu et al., 2014). The cell-associated matriptase-HAI-1 complex will be shed into the extracellular milieu hours after zymogen activation induced (Tseng et al., 2010).

**Fig. 6.**
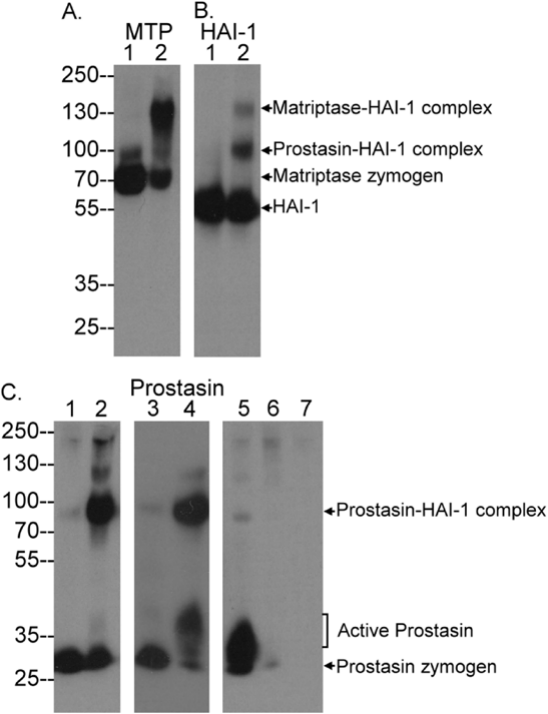
**Human keratinocytes retain active prostasin.** HaCaT human keratinocytes were transiently exposed to PBS as a non-activation control (lanes 1 in A,B; lanes 1 and 3 in C) or a pH 6.0 buffer to induce zymogen activation of matriptase and prostasin (lanes 2, in A,B; lanes 2, 4, and 5 in C). A separate sample of the lysate used for lane 5 was subjected to immunodepletion of prostasin using the mAb YL11-conjugated Sepharose and analyzed in lane 6 (lane 6 in C). The conditioned buffer following the induction of zymogen activation was collected and concentrated to the volume equal to that of the cell lysate (lane 7). These samples were analyzed for the species of matriptase (A, MTP), HAI-1 (B, HAI-1) and prostasin (C). The identity of the protein bands are as indicated.

We used the mildly acidic buffer to induce the zymogen activation of both proteases in order to compare the fate of active matriptase and active prostasin. As described previously and observed in the current study (Fig. 6), on transient exposure of HaCaT cells to a pH 6.0 buffer the 70-kDa matriptase zymogen was rapidly converted into its activated form. A vast majority of activated matriptase are rapidly inhibited by HAI-1 via formation of a 120-kDa activated matriptase-HAI-1 complex and remain cell-associated, which can be shown by immune-blot analysis of lysates prepared from control- and acid-exposed cells (Fig. 6A, comparing lane 2 to lane 1). The 120-kDa matriptase-HAI-1 complex was also detected by a HAI-1 mAb (Fig. 6B, lane 2).

When the lysates from control and pH 6.0 exposed cells were analyzed for prostasin-containing species, the 100-kDa prostasin-HAI-1 complex was the major product detected after the induction of zymogen activation (Fig. 6C, comparing 2 to lane 1). The size of the prostasin-HAI-1 complex has previously been described as being 85-kDa, but this difference in apparent size is due to differences in the molecular weight markers used (Chen et al., 2010b). The prostasin-HAI-1 complex can be detected by the prostasin mAb (Fig. 6C, comparing lane 2 to lane 1) and the HAI-1 mAb (Fig. 6B, lane 2). Free active prostasin was also detected with a size slightly greater than prostasin zymogen under non-reducing and non-boiled conditions. The identification and characterization of the free active prostasin can be found in our previous study (C.-Y.L. unpublished data). The levels of free active prostasin (Fig. 6C) and the ratio between free active prostasin and prostasin zymogen appear to vary significantly from experiment to experiment. In one experiment, almost all activated prostasin was detected in complex with HAI-1, and free active prostasin was barely detected (Fig. 6C, lane 2). In other experiments, however, active prostasin was detected at significant levels (Fig. 6C, lane 4), or indeed represented the vast majority of activated prostasin (Fig. 6C, lane 5). The identity of the three bands tentatively identified as prostasin species by western blot analysis was confirmed by the loss of these bands following immunodepletion of the lysates using prostasin mAb beads (Fig. 6C, lane 6). While significant activated prostasin was detected in cell lysates, no shed prostasin was detected in the conditioned buffer following induction of zymogen activation of matriptase and prostasin (Fig. 6C, lane 7). Collectively, while both active matriptase and active prostasin can be inhibited by HAI-1, cell-associated free active prostasin appears to persist for some time, in contrast to active matriptase which is rapidly inhibited by HAI-1 when cell-associated, and can only escape HAI-1-mediated inhibition if it is rapidly shed into extracellular milieu (Chen et al., 2010b; Chu et al., 2014).

## DISCUSSION

The epidermis, hair follicle, and sebaceous gland resemble each other with respect to their capacity of constant self-renewal throughout life. This self-renewal begins with the proliferation of stem cells followed by formation of the committed cells through the process of differentiation. This is a highly regulated process and so it is not surprising that a consistent distribution profile across the three skin compartments can be observed for the expression of matriptase, prostasin, or HAI-1 over the course of differentiation. The expression of high levels of matriptase but negligible levels of prostasin in the basal and basaloid cells of the human epidermis and pilosebaceous unit suggests that human matriptase contributes to proliferation and early differentiation of the skin (Chen et al., 2013a) and that these processes likely do not involve prostasin. In these cells, matriptase can undergo autoactivation to acquire its enzymatic activity in the absence of prostasin. Similarly, the expression of prostasin in the differentiated cells with little or no matriptase expression suggests that prostasin may regulate the late stages of differentiation and epidermal barrier function in these cells in the absence of matriptase as upstream activator (Leyvraz et al., 2005). In spite of their apparently different functioning in proliferation and the late stages of differentiation, matriptase and prostasin do share the same inhibitor mechanism through the formation of a very stable and tightly bound complex with HAI-1, which is broadly expressed.

The shared HAI-1 inhibitory mechanism and the tightly coupled zymogen activation observed in HaCaT cells was highly suggestive that matriptase and prostasin might function as a tightly coupled protease cascade, whereby contributing to the regulation of proliferation and early differentiation in human skin but not to the late stages of differentiation in mouse skin. It is, therefore, surprising to observe the inverse trend for expression over the course of differentiation for matriptase in contrast to that for prostasin. The difference in subcellular targeting could in part be the reason for the apparent functional disconnect between the proteins, at least in human skin. Matriptase has long been known for its expression at the basolateral plasma membrane in simple epithelium, such as in the kidney and prostate (Wang et al., 2009). It is conceivable and consistent with its *in vivo* basolateral localization that matriptase is expressed at the cell-cell junctions in stratified epithelium in human skin and cultured keratinocytes. Although prostasin and matriptase are both membrane-associated, the mechanism for their anchorage at the lipid bilayer membrane is different. Prostasin is a GPI-anchored protein (Chen et al., 2001), and the vast majority of GPI-anchored proteins are targeted to the apical plasma membrane of simple epithelium. Detection of prostasin primarily in the membrane protrusion, rather than the cell-cell junctions of HaCaT keratinocytes, could simply be a manifestation of the intrinsic tendency for a GPI-anchored protein targeting to the sub-domain of plasma membrane in the absence of contact with neighboring cells, like the apical plasma membrane. The detection of prostasin in polarized patches, rather than the entire cell-cell contacts, in the upper spinous layers in the epidermis could also result from the intrinsic tendency of membrane targeting. The different subcellular localizations of the proteases make it difficult for direct interaction between matriptase and prostasin to occur. The significant organelle re-organization at the late stages of keratinocyte differentiation could change the default destinations of matriptase and prostasin in the secretory pathway and bring the two serine proteases together. If organelle re-organization is the mechanism for the functional link between matriptase and prostasin, it must only occur in mouse skin, and not in human skin, in which matriptase is simply not expressed by the differentiated cells. The differences in matriptase distribution in human versus mouse skin are not completely surprising. Mouse skin resembles the human counterpart in many ways and has been widely used as a model system to study the biology and pathogenesis of skin. Significant differences in the anatomical and physiological aspects of the tissue in mouse versus human have, however, been described, including the thickness and layers of the epidermis, the density of hair follicles, the presence and role of subcutaneous panniculus carnosus muscle in wound healing, the synchronism of the hair follicle cycle (Stenn and Paus, 2001), the rate of epidermis turnover (Berking et al., 2002), and the efficiency in epidermal barrier function and percutaneous absorption (Menon, 2002). These significant anatomical and physiological differences appear to result in, and are consistent with, the significant difference in the expression of skin-associated genes between human skin and mouse skin (Gerber et al., 2014). It is worth noting that the evidence which suggested a functional link exists between matriptase and prostasin was the observed lack of prostasin zymogen activation in the skin of matriptase knockout mice, in addition to the similarity in the epidermal defects associated with genetic ablation of matriptase and prostasin (Netzel-Arnett et al., 2003). The assessment of the state of prostasin zymogen activation was made based on the relative levels of prostasin species of different sizes in the skin of matriptase knockout mice; specifically the disappearance of a species of smaller size in the knockout mice. This smaller prostasin species was reported to be the activated form of prostasin, since the cleavage of the zymogen during activation would result in the loss of the 12 amino acid light chain, thereby resulting in a smaller prostasin species observed by SDS-PAGE under reducing and boiled conditions. It is not clear, however, that this interpretation of the data is correct, since it seems unlikely that SDS-PAGE has adequate resolution to detect the loss of 12 amino acids and distinguish the activated from the zymogen forms of prostasin (Friis et al., 2013). Thus, the small prostasin species that is lost in the matriptase knockout mice may very well not represent the active form of the enzyme since the size difference seems too big to be explained by the loss of 12 amino acids. Indeed, multiple active prostasin species of different sizes isolated from human semen can be detected by SDS-PAGE analysis, and are apparently generated during synthesis and maturation (Yu et al., 1994). Differential N-glycosylation, and other less well characterized C-terminal processing events, are the likely culprit. Given the findings of the current study, and the potential limitations of the mouse study mentioned above, the question of whether matriptase ablation causes the loss of prostasin zymogen activation in mouse skin should probably be revisited.

The release of matriptase and/or prostasin, particularly in their active forms, from the cell membrane could provide a means for their interaction. Following the induction of robust zymogen activation in HaCaT cells, active matriptase, but not active prostasin, is shed into the extracellular milieu. Although the shed active matriptase could return to the surface of the HaCaT cells to activate prostasin, it remains unclear whether this is a relevant mechanism *in vivo* in human skin. Matriptase zymogen activation *in vivo* likely does not occur to the same levels observed in HaCaT cells exposed to a mildly acidic buffer due to the presence of sodium chloride in the skin. Chloride ions can significantly attenuate acid-induced matriptase zymogen activation and thereby reduce the level of active matriptase shed (Wang et al., 2014). In fact, matriptase zymogen activation in human epidermis is rather weak and mainly focused on the basal cells, a situation similar to that observed in hair follicles (Fig. 2). Furthermore, any active matriptase shed from the basal cells is likely inhibited by HAI-1 resided on the surface of spinous cells before it could reach the prostasin zymogen at the upper spinous layers. Although matriptase and prostasin may still have a functional relationship in some way not yet understood, the inverse expression pattern during the course of keratinocyte differentiation indicates that the primary physiological functions of the two serine proteases are different.

In summary, matriptase and prostasin are differentially expressed at different stages of differentiation in human epidermis and pilosebaceous unit. The lack of co-expression in the proliferative or differentiated cells makes the two membrane-associated serine proteases less likely to function as a tightly coupled proteolytic cascade. Matriptase and prostasin participate in the regulation of proliferation and late-stage differentiation, respectively, as independent entities in the absence of functional link, at least at the zymogen activation level. The loose and limited functional link between matriptase and prostasin in human skin is in stark contrast to their close functional relation in mouse skin.

## MATERIAL AND METHODS

### Reagents

Alexa Fluor 594 goat anti-mouse IgG, Alexa Fluor 488 goat anti-mouse, Alexa Fluor 594 phalloidin, and Alexa Fluor 488 phalloidin were obtained from Molecular Probes (Eugene, Oregon). 5,5′-Dithio-bis-(2-Nitrobenzoic Acid) (DTNB) and 4′,6-diamidino-2-phenylindole (DAPI) were obtained from Sigma-Aldrich (St. Louis, MO). Horseradish peroxidase (HRP)-conjugated secondary antibodies were purchased from Kirkegaard & Perry Laboratories (Gaithersburg, MD). Western Lightning Chemiluminescence Reagent Plus was purchased from PerkinElmer Life Sciences (Waltham, MA).

### Cell cultures

HaCaT human keratinocytes (CLS Cell Lines Service GmbH, Eppelheim Germany), were cultured in DMEM supplemented with 10% fetal bovine serum (FBS). The cells were incubated at 37°C in a humidified atmosphere with 5% CO_2_.

### Antibodies

The total matriptase monoclonal antibodies (mAbs) M24 and M32, activated matriptase mAb M69, and HAI-1 mAb M19 were generated using matriptase-HAI-1 complex as the immunogen and characterized as previously described (Chen et al., 2010b; Lin et al., 1997, 1999; Tseng et al., 2010). The prostasin mAb YL11 was generated using matriptase-HAI-1 complex as the immunogen, as described previously (Lai et al., 2016). YL11 mAb-Sepharose was generated by conjugating the mAb to CNBr-activated Sepharose 4B (at 5 mg/ml gel), based on the manufacturer's instruction (GE Healthcare, Uppsala, Sweden).

### Immunohistochemistry

The skin tissue sections were obtained with written informed consent from Tri-Service General Hospital, National Defense Medical Center under IRB 099-05-019, approved by Tri-Service General Hospital Internal Review Board (TSGHIRB). Immunohistochemical staining was performed as previously described (Chen et al., 2011, 2010a). Tissue sections of frozen human skin were fixed with formalin and stained using the total matriptase mAb M32, activated matriptase mAb M69, prostasin mAb YL11, HAI-1 mAb M19, or mouse IgG as negative control, followed by the secondary antibody (EnVision+ Dual Link System Peroxidase) (Dako, Glostrup, Denmark). DAB (3, 3′-Diaminobenzidine) was used for the detection of positive staining. Cell nuclei were counterstained with hematoxylin. Images were captured using an Olympus AH2 Vanox Microscope System (Olympus, Melville, NY). Studies demonstrating the specificity of these mAbs and describing their application in IHC staining can be found in our previous studies (Benaud et al., 2001; Chen et al., 2011; Lin et al., 1999; Tseng et al., 2010; Wang et al., 2009).

### Acid-induced zymogen activation of matriptase and prostasin

HaCaT human keratinocytes were washed with PBS three times and then incubated with 150 mM phosphate buffer pH 6.0 at room temperature for 20 min as previously described (Lee et al., 2007; Tseng et al., 2010). The supernatant was collected as the conditioned buffer which contained the shed active matriptase (Chen et al., 2013a,b). The cells were then washed with PBS once and lysed in 1% Triton X-100, 1 mM DTNB in PBS for immunoblot analysis.

### Western blotting

HaCaT human keratinocytes were lysed in PBS containing 1% Triton X-100 and 1 mM DTNB (Lee et al., 2005). The cell lysates and the concentrated conditioned buffer were diluted with 5× SDS sample buffer containing no reducing agent and incubated at room temperature for 5 min prior to loading onto the gels. Proteins were resolved by 7.5% SDS-PAGE, transferred to nitrocellulose membranes, and probed with the indicated mAbs. The binding of the mAbs was detected using HRP conjugated secondary antibodies, and visualized using Western Lightening Chemiluminescence Reagent Plus (Perkin-Elmer, Boston, MA).

### Immunodepletion

The prostasin mAb YL11 was covalently coupled to Sepharose 4B at 5 mg/ml gel as described previously (Xu et al., 2012). For immunodepletion, the cell lysates (200 µl) were incubated with 15 µl of the mAb conjugated Sepharose in micro-centrifuge tubes that were rotated end over end in a cold room for 2 h. The supernatants were collected by centrifugation as the immunodepleted fraction.
